# Synthesis and evaluation of a novel analgesic conotoxin Lt7b that inhibits calcium currents and increases sodium currents

**DOI:** 10.1111/jcmm.17521

**Published:** 2022-09-01

**Authors:** Yun Wu, Manyi Yang, Yubin Li, Wei Zhang, Maojun Zhou

**Affiliations:** ^1^ Guangdong Provincial Key Laboratory of Medical Molecular Diagnostics, The First Dongguan Affiliated Hospital Guangdong Medical University Dongguan China; ^2^ Department of Hepatobiliary and Pancreatic Surgery, NHC Key Laboratory of Nanobiological Technology Xiangya Hospital, Central South University Changsha China; ^3^ Department of Oncology, NHC Key Laboratory of Cancer Proteomics, State Local Joint Engineering Laboratory for Anticancer Drugs Xiangya Hospital, Central South University Changsha China; ^4^ Institute of High Energy Physics Chinese Academy of Sciences Shijingshan District China

**Keywords:** calcium channel blockers, conopeptide, conotoxin, *conus literatus*, Lt7b, pain, sodium currents

## Abstract

Conotoxins are promising neuropharmacological tools and drug candidates due to their high efficiency and specificity in targeting ion channels or neurotransmitter receptors. In this study, a novel O_2_‐superfamily conotoxin, Lt7b, was synthesized and its pharmacological functions were evaluated. Lt7b with three modified amino acids and three disulfide bonds was successfully synthesized. CD spectra showed that Lt7b had a typical α‐helix in the secondary structure. Patch clamp experiments on rat DRG neurons showed that Lt7b could significantly inhibit calcium currents with an IC_50_ value of 856 ± 95 nM. Meanwhile, 10 μM Lt7b could significantly increase the sodium currents by 77 ± 8%, but it had no obvious effects on the potassium currents in DRG neurons. In addition, patch clamp experiments on ion channel subtypes showed that 10 μM Lt7b could inhibit 7.0 ± 1.2%, 8.0 ± 1.5%, 4.6 ± 3.4%, and 9.5 ± 0.1% of the hCa_v_1.2, hCa_v_2.1, hCa_v_2.2, and hCa_v_3.2 currents, respectively, while it did not increase the rNa_v_1.7, rNa_v_1.8, hNa_v_1.5, hNa_v_1.7, and hNa_v_1.8 currents. Lt7b had no obvious toxicity to HaCaT and ND7/23 cells up to 1 mM and significantly increased the pain threshold at the testing time of 0.5–4 h in a dose‐dependent manner in the mouse hotplate assay. This novel conotoxin Lt7b may be a useful tool for ion channel studies and analgesic drug development.

## 1 INTRODUCTION

Conotoxins are promising neuropharmacological tools and drug candidates due to their high efficiency and specificity in targeting ion channels or neurotransmitter receptors.^1^, ^2^ To date, 2986 nucleic acid sequences, 8123 protein sequences, and 222 structures of conotoxins have been collected by ConoServer.^3^ One conotoxin, ω‐conotoxin MVIIA (Ziconotide), was approved by the FDA for treating intractable chronic pain in 2004, and several other conotoxins were under clinical or preclinical phases for the treatment of Alzheimer, Parkinson, epilepsy, chronic pain, and cardiovascular diseases.^4^, ^5^, ^6^

In this work, we reported the synthesis and pharmacological functions of a novel O_2_‐superfamily conotoxin Lt7b. Patch clamp tests on rat DRG cells and HEK293 cells were used to test the inhibitory effects of Lt7b on calcium and sodium currents. Moreover, the animal analgesia experiments were conducted by the hotplate assay.

## 2 MATERIALS AND METHODS

Detailed materials and methods are provided in the Appendix S1.

## 3 RESULTS

### 3.1 Synthesis and identification of Lt7b

Conotoxins of the same superfamily usually have similar disulfide bonding and posttranslational modifications. According to the sequence analysis of the known O_2_‐superfamily conotoxins, we speculated that Lt7b might have three posttranslationally modified amino acids similar to other O_2_‐superfamily conotoxins (Table S1), so we replaced three original amino acid residues of Lt7b with modified residues: 11P (proline in the eleven position) was replaced by hydroxyproline (O); 13E and 20E were replaced by γ‐carboxyglutamate (γ). Referring to the disulfide linkage of other O_2_‐superfamily toxins, we speculated that the disulfide bond pattern is 1–4, 2–5, and 3–6. The final synthetic sequence of Lt7b is **C**TDWLGS**C**SS**O**S**γCC**YDN**Cγ**TY**C**TLWK (1–15, 8–19, and 14–23).

The conotoxin peptide Lt7b was synthesized on a Rink amide resin using a standard Fmoc strategy (Figure S1). The oxidized peptide was synthesized with three modified amino acids and three disulfide bonds. The oxidized peptide was then purified by RP‐HPLC (Figure S2A), and the molecular weight was confirmed by mass spectrometry (Figure S2B). The mass of the oxidized peptide was 3196.0 Da, which was consistent with the expected mass, suggesting that Lt7b with three modified amino acids and three disulfide bonds was successfully synthesized. CD spectra showed that Lt7b has a positive peak at approximately 190 nm, indicating that there was a typical α‐helix in the secondary structure of Lt7b (Figure S2C).

### 3.2 Effects of Lt7b on DRG sodium, potassium, and calcium currents

**FIGURE 1:**
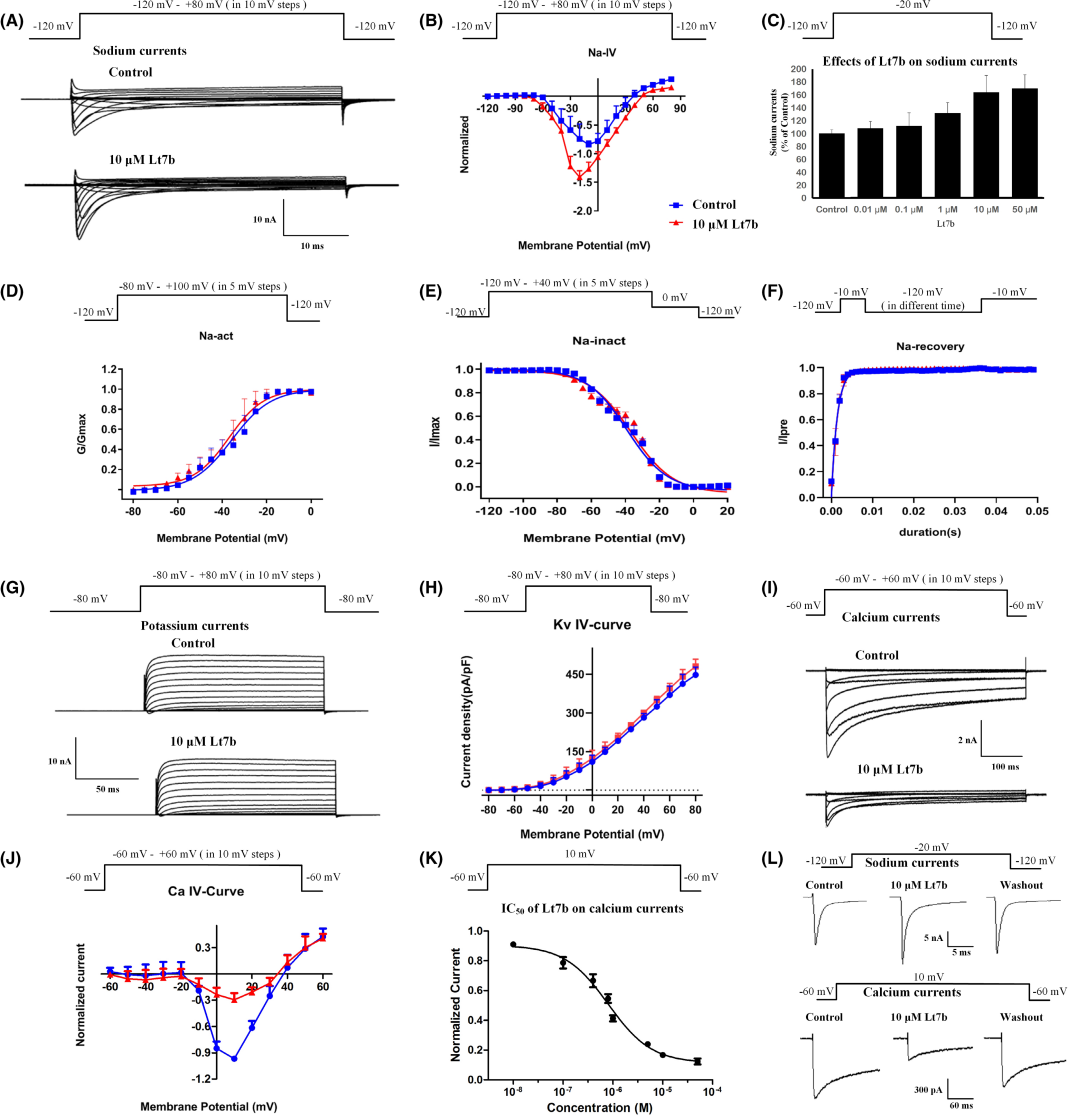
Effects of Lt7b on DRG sodium, potassium, and calcium currents. (A) Effects of 10 μM Lt7b on sodium currents in rat DRG neurons. Effects of 10 μM Lt7b on the current–voltage (I–V) relationships (B), concentration‐response relationship (C), activation (D), inactivation (E), and recovery (F) of sodium currents in DRG neurons. (G) Effects of 10 μM Lt7b on potassium currents in rat DRG neurons. (H) Effects of 10 μM Lt7b on the current–voltage (I–V) relationships of potassium currents in DRG neurons. (I) Effects of 10 μM Lt7b on calcium currents in rat DRG neurons. (J) Effects of 10 μM Lt7b on the current–voltage (I–V) relationships of calcium currents in DRG neurons. (K) IC_50_ value of Lt7b on calcium currents in rat DRG neurons. (L) Washout of 10 μM Lt7b on sodium and calcium currents in rat DRG neurons

Lt7b was tested for its effects on sodium, potassium, and calcium currents in the acute isolated rat DRG neurons using patch clamp. For sodium currents, medium DRG neurons (diameters 20–30 μm) were used to record TTX‐sensitive and TTX‐resistant mixed sodium currents. 10 μM Lt7b could significantly increase the mixed sodium currents (Figure 1A), and the peak sodium currents were enhanced by 77 ± 8% (Figure 1B, *n* = 3). The effects of Lt7b (0.01, 0.1, 1, 10, and 50 μM) on sodium currents showed that SIIID increased sodium currents in a concentration‐dependent manner (Figure 1C). 10 μM Lt7b had no obvious effects on the activation (Figure 1D, *n* = 3), inactivation (Figure 1E, *n* = 3), or recovery (Figure 1F, *n* = 3) of the sodium currents in rat DRG neurons, and the detailed fit parameters are shown in Figure S3. For the potassium currents, 10 μM Lt7b had no obvious effects on the potassium currents (Figure 1G) and did not induce a shift in the current–voltage relationship (Figure 1H, *n* = 3).

For the calcium currents in rat DRG neurons, 10 μM Lt7b could significantly inhibit the calcium currents (Figure 1I), and the peak calcium currents were reduced 75 ± 8% (Figure 1J, *n* = 3). 10 μM Lt7b did not induce a shift in the current–voltage relationship (Figure 1J). The IC_50_ value of Lt7b on calcium currents in rat DRG neurons was 856 ± 95 nM (Figure 1K, *n* = 3). In addition, washout recovered the sodium and calcium currents (Figure 1L), indicating that the effects of Lt7b on sodium and calcium currents were reversible.

### 3.3 Effects of Lt7b on sodium and calcium channel subtypes

**FIGURE 2:**
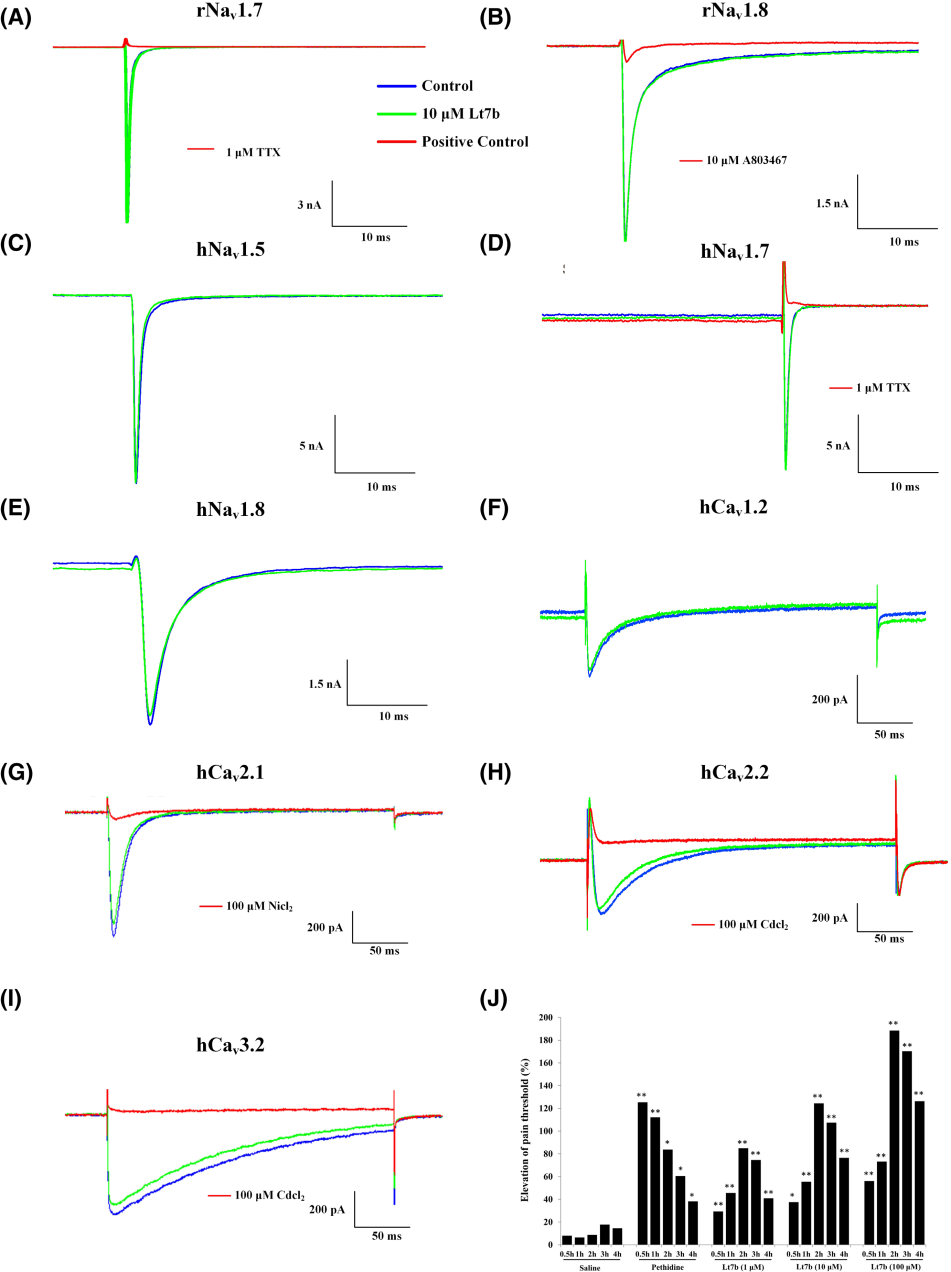
Effects of Lt7b on ion channel subtypes and analgesic effects in the mouse hotplate assay. (A) Effects of 10 μM Lt7b on rNa_v_1.7 (A), rNa_v_1.8 (B), hNa_v_1.5 (C), hNa_v_1.7 (D), hNa_v_1.8 (E), hCa_v_1.2 (F), hCa_v_2.1 (G), hCa_v_2.2 (H), and hCa_v_3.2 (I) in HEK293 cells. 1 μM TTX was used as a positive control in rNa_v_1.7 and hNa_v_1.7 experiments. 10 μM A803467 was used as a positive control in rNa_v_1.8 experiments. 100 μM NiCl_2_ was used as a positive control in hCa_v_2.1 experiments. 100 μM CdCl_2_ was used as a positive control in hCa_v_2.2 and hCa_v_3.2 experiments. (J) Analgesic effects of Lt7b tested by the mouse hotplate assay. The relationship between test time and the increased percentage of pain threshold (%) was shown. *p < 0.01, **p < 0.001

Plasmids of rNa_v_1.7 (TTX‐sensitive), rNa_v_1.8 (TTX‐resistant), hNa_v_1.5 (TTX‐resistant), hNa_v_1.7 (TTX‐sensitive), hNa_v_1.8 (TTX‐resistant), hCa_v_1.2, hCa_v_2.1, hCa_v_2.2, and hCa_v_3.2 were transfected into HEK293 cells, and Lt7b was tested on these sodium and calcium channel subtypes. For the sodium channel subtypes, 10 μM Lt7b had no obvious effects on the rNa_v_1.7 (Figure 2A), rNa_v_1.8 (Figure 2B), hNa_v_1.5 (Figure 2C), and hNa_v_1.7 (Figure 2D) currents. 10 μM Lt7b could inhibit 4.6 ± 0.8% of the hNa_v_1.8 currents (Figure 2E, *n* = 3). For the calcium channel subtypes, 10 μM Lt7b could inhibit 7.0 ± 1.6% of the hCa_v_1.2 currents (Figure 2F, *n* = 3), 8.0 ± 1.5% of the hCa_v_2.1 currents (Figure 2G, *n* = 3), 4.6 ± 3.4% of the hCa_v_2.2 currents (Figure 2H, *n* = 3), and 9.5 ± 0.1% of the hCa_v_3.2 currents (Figure 2I, *n* = 3).

### 3.4 The cytotoxicity of Lt7b

To determine the cytotoxicity of Lt7b, the viability of HaCaT and ND7/23 cells incubated with different concentrations of Lt7b was measured by MTT (Table S2). The cell viability values were more than 96% at all detected concentrations (0.01, 0.1, 1, 10, 100, and 1000 μM), indicating that Lt7b had no significant cytotoxicity against HaCaT and ND7/23 cells up to 1 mM.

### 3.5 The analgesic activity of Lt7b

The analgesic activity of Lt7b was evaluated by the mouse hotplate assay, which was tested at 0.5, 1, 2, 3, and 4 h after intrathecal injection (Figure 2J). Pethidine (10 mM) was used as a positive control in this experiment. In the pethidine group, the analgesic effects reached a maximum at 0.5 h, with the pain threshold increasing 125.46% and then decreasing over time. All three doses of Lt7b increased the pain threshold at the testing time of 0.5–4 h. The analgesic effect of the high‐dose group of Lt7b (100 μM) reached a maximum at 2 h, and the pain threshold increased 188.47%. At 0.5 and 1 h, pethidine showed better analgesic effects than Lt7b, while at 2, 3, and 4 h, Lt7b showed better analgesic effects than pethidine.

## 4 DISCUSSION

To date, four of the seven reported O_2_‐superfamily conotoxins have shown significant functional diversity (Table S1).^7^, ^8^ TxVIIA and PnVIIA are agonists of neuronal pacemaker cation currents; Lt7a blocks voltage‐sensitive sodium channels; PiVIIA increases Ca^2+^ currents. The functions of As7a and De7a are still unknown and might also affect voltage‐gated nonspecific cation pacemaker channels.^9^, ^10^ In this study, the novel O_2_‐conotoxin Lt7b with three modified amino acids and three disulfide bonds was successfully synthesized. Patch clamp experiments showed that Lt7b inhibited calcium currents and increased sodium currents in rat DRG neurons.

The ω‐conotoxins selectively block the voltage‐gated calcium channel (Ca_v_2.2), leading to their development as intrathecal analgesics for severe pain.^11^ One of these calcium channel inhibitors, ω‐conotoxin MVIIA, was approved by the FDA for treating intractable chronic pain in 2004.^6^ Similar to the ω‐conotoxins, Lt7b inhibited the calcium currents in rat DRG neurons with an IC_50_ value of 856 ± 95 nM and showed analgesic activities in the mouse hotplate assay. Unfortunately, Lt7b had slight inhibitory effects on human Ca_v_1.2, Ca_v_2.1, Ca_v_2.2, and Ca_v_3.2 currents. Whether Lt7b could inhibit other human calcium channel subtypes should be studied in future research.

In conclusion, this study identifies the function of a novel O_2_‐superfamily conotoxin, Lt7b, with a typical α‐helical structure. Lt7b could significantly inhibit calcium currents and had analgesic effects. This novel conotoxin Lt7b may be a useful tool for studying calcium channels and developing analgesic drugs.

## AUTHOR CONTRIBUTIONS

**Yun Wu:** Data curation (equal); funding acquisition (equal); investigation (lead); writing – original draft (equal). **Manyi Yang:** Data curation (equal); investigation (lead); writing – original draft (equal). **Yubin Li:** Investigation (supporting). **Wei Zhang:** Investigation (supporting). **Maojun Zhou:** Conceptualization (equal); data curation (equal); formal analysis (equal); funding acquisition (equal); investigation (lead); writing – original draft (equal); writing – review and editing (equal).

## Funding information

This research was funded by the National Natural Science Foundation of China (81703412), the Natural Science Foundation of Hunan Province (2021JJ30914 and 2022JJ30974), the Guangdong Basic and Applied Basic Research Foundation (2020A1515111040), the Medical Scientific Research Foundation of Guangdong Province (B2021197), and the Foundation for Distinguished Young Talents in Higher Education of Guangdong (2018KQNCX089).

## CONFLICT OF INTEREST

The authors declare no conflicts of interest.

## Supporting information


Appendix S1

## Data Availability

The data that support the findings of this study are available from the corresponding author upon reasonable request.
