# Supplementary material for: Synthesis and evaluation of a novel analgesic conotoxin Lt7b that inhibits calcium currents and increases sodium currents
Source: J Cell Mol Med. 2022 Sep 1;26(20):5330–4. doi: 10.1111/jcmm.17521 (PMC9575111; doi:10.1111/jcmm.17521)
Supplement: Supplementary file 1 — Appendix S1 [file JCMM-26-5330-s001.docx]

**Appendix S1**

**Supporting Information**

**1. Materials and Methods**

**1.1 Synthesis of peptide Lt7b**

Conotoxin Lt7b (GenBank number: DQ512967.1), whose mature amino acid sequence is CTDWLGSCSSOSγCCYDNCγTYCTLWK, was synthesized by solid-phase polypeptide synthesis. O indicates hydroxyproline and γ indicates γ-carboxyglutamate. The three disulfide bonds are 1-15, 8-19, and 14-23. Briefly, conotoxin peptide was assembled on Rink-resin using the Fmoc-strategy according to its amino acid sequence. The amino acid residues were coupled using HOBt/HBTU/DIPEA (1:1:0.9) for 2 h and cleaved from the resin with regent R (90% TFA: 5% thioanisole: 3% 1,2-ethanedithiol: 2% anisole). The three pairs of cysteines were protected by acetamidomethyl (Acm), methoxytriphenyl (Mmt) or triphenylmethyl (Trt) separately, and the three disulfide bonds were successively formed by oxidation. After oxidation, the mature peptide was purified through a C18 reverse phase column by reversed-phase high-performance liquid chromatography (RP-HPLC), and the molecular weight was confirmed by mass spectrometry analysis. After purification by HPLC, the purity of synthetic Lt7b was more than 98%.

**1.2 Circular dichroism measurement**

Circular dichroism (CD) spectra were measured by a Chirascan spectropolarimeter instrument (Applied Photophysics, England). The purified Lt7b was dissolved in PBS buffer to a final concentration of 0.1 mg/mL. The spectra were recorded over a 180-300 nm range at 20 °C using an average of 5 scans (scan speed 100 nm/min). The percentages of protein secondary structures were estimated using a Kohonen neural network with a 2-dimensional output layer by DicroProt.

**1.3 Whole-cell patch clamp for DRG cells**

The study was conducted in accordance with the Laboratory Animal-Guideline for Ethical Review of Animal Welfare (GB/T 35892-2018, China). Sprague–Dawley rats (30 days old) were purchased from Guangzhou University of Chinese Medicine Experimental Animal Center (NO. SYXK(Yue)2018-0182). All animal procedures were carried out according to the approved protocol (GDY2002208) of the Institutional Animal Care and Use Committee at Guangdong Medical University. The rats were euthanized, and the dorsal root ganglia tissue was removed quickly and cut into small pieces. The ganglia were treated with 0.1% collagenase and 0.05% trypsin. After centrifugation, the DRG cells were suspended in essential DMEM with 10% (v/v) fetal bovine serum and incubated in a 5% CO_2_ fully humidified environment at 37 °C.

Medium DRG neurons (diameters 20–30 μm) were used to record TTX-sensitive and TTX-resistant mixed sodium currents. To acquire current-voltage (I-V) relationships of sodium channels in DRG cells, test potentials ranged from -120 to +80 mV in 10 mV steps from a holding potential of -120 mV using EPC-10 (HEKA, Germany). For the activation curve of sodium channels, test potentials ranged from -80 to +0 mV in 5 mV steps from a holding potential of -120 mV. For the inactivation curve of sodium channels, test potentials ranged from -120 to +20 mV in 5 mV steps from a holding potential of -120 mV for 1000 ms, then stepped to 0 mV for 50 ms, and finally stepped to -120 mV for 150 ms. For the recovery curve of sodium channels, test potential was stepped to -10 mV for 5 ms to inactivate the sodium current, then stepped to -120 mV for different times to allow the sodium current to recover, and finally stepped to -10 mV for 50 ms to detect the sodium current.

To acquire current-voltage (I-V) relationships of potassium channels in DRG cells, test potentials ranged from -80 to +80 mV in 10 mV steps from a holding potential of -80 mV using EPC-10 (HEKA, Germany). To acquire current-voltage (I-V) relationships of calcium channels in DRG cells, test potentials ranged from -60 to +60 mV in 5 mV steps from a holding potential of -60 mV. All the patch clamp data were analyzed by SigmaPlot. The concentration–response curve was fitted using the following Hill equation: y=1-(1-fmax)/[1+([S]/IC_50_)n], where S is the Lt7b concentration, n is the empirical Hill coefficient, and fmax is the maximum calcium current inhibition rate (I/Imax, where Imax is the control peak of calcium current).

**1.4 Whole-cell patch clamp for HEK293 cells**

HEK293 cells, which were obtained from the Cell Bank of the Chinese Academy of Sciences (Shanghai, China) with STR Authentication, were cultured in DMEM with 10% fetal bovine serum. Plasmids of rat Na_v_1.7 (rNa_v_1.7), rat Nav1.8 (rNa_v_1.8, cotransfected with Na_v_β1), human Na_v_1.5 (hNa_v_1.5), human Na_v_1.7 (hNa_v_1.7), human Na_v_1.8 (hNa_v_1.8, cotransfected with Na_v_β1), human Ca_v_1.2 (hCa_v_1.2), human Ca_v_2.1 (hCa_v_2.1), human Ca_v_2.2 (hCa_v_2.2) and human Ca_v_3.2 (hCa_v_3.2) were cloned by our lab and were separately transfected into HEK293 cells using Lipofectamine 3000 (Invitrogen, America) as previously described ^1, 2^. The sodium currents were induced by a depolarization of -10 mV from a holding potential of -80 mV. The calcium currents of hCa_v_1.2, hCa_v_2.1, hCa_v_2.2 and hCa_v_3.2 were induced by a 400 ms depolarization of 10 mV from a holding potential of -60 mV.

**1.5 MTT cytotoxicity assay**

HaCaT cells were purchased from the Beijing Biobw Co. Ltd (Beijing, China). ND7/23 cells were obtained from the National Collection of Authenticated Cell Cultures (Shanghai, China). The cytotoxic activities of conotoxin Lt7b were examined against HaCaT and ND7/23 cells. HaCaT cells were grown in DMEM high glucose medium containing 2 mM L-glutamine and 10% (v/v) fetal bovine serum, 100 μg/mL penicillin and 100 μg/mL streptomycin in a 5% CO_2_ fully humidified environment at 37 °C. ND7-23 cells were grown in DMEM high glucose growth medium supplemented with 10% fetal bovine serum, 1% Glutamax, 50 U/mL penicillin and 50 µg/mL streptomycin at 37 °C with a 5% CO_2_/95% air humidified atmosphere. A cytotoxicity assay was carried out in vitro using MTT staining. The peptide concentrations used were 0, 0.01, 0.1, 1, 10, 100 and 1000 μM for each well, respectively. Three separate experiments were carried out, and six replicate wells were used to determine each point. After 48 h of incubation, the cells were stained with MTT and placed in a BIO-RAD model 680 microplate reader to determine the absorbance at 490 nm.

**1.6 Analgesic activity bioassays**

Female Kunming mice (weight 18–22 g) were purchased from Guangzhou University of Chinese Medicine Experimental Animal Center (NO. SYXK(Yue)2018-0182). All animal procedures were carried out according to the approved protocol (GDY2002208) of the Institutional Animal Care and Use Committee at Guangdong Medical University. Thirty Kunming mice were randomly divided into five groups. Each mouse was intrathecally injected with 10 μL Lt7b (1, 10, 100 μM), pethidine (positive control, 10 mM, which is 1.25 mg/kg), or 0.9% saline (negative control). The pain threshold was set as the time from mice on the 55 °C hotplate to licking foot. The pain threshold was tested at 12 h before drug administration and 0.5 h, 1 h, 2 h, 3 h, and 4 h after drug administration. Pain threshold increment percentage (%) = (pain threshold after administration - pain threshold before administration)/pain threshold before administration × 100%.

**1.7 Statistical analysis**

*P* values were calculated with the Student’s t-test. *P* < 0.05 was considered to be statistically significant and *P* values are designated as follows: **P* < 0.05; ***P* < 0.01. All error bars in the graphs represent the standard error of the mean calculated from at least three replicates.

**References**

1. Manyi Yang, Yubin Li, Longfei Liu, Maojun Zhou , A novel M-superfamily proline-rich conotoxin that can simultaneously affect sodium, potassium and calcium currents. J Venom Anim Toxins incl Trop Dis, 2021, 27: e20200164.

2. Yang, M., Zhou, M. μ-conotoxin TsIIIA, a peptide inhibitor of human voltage-gated sodium channel hNav1.8, Toxicon, 2020.8.3, 186: 29~34.

**2. Supplementary results**

**
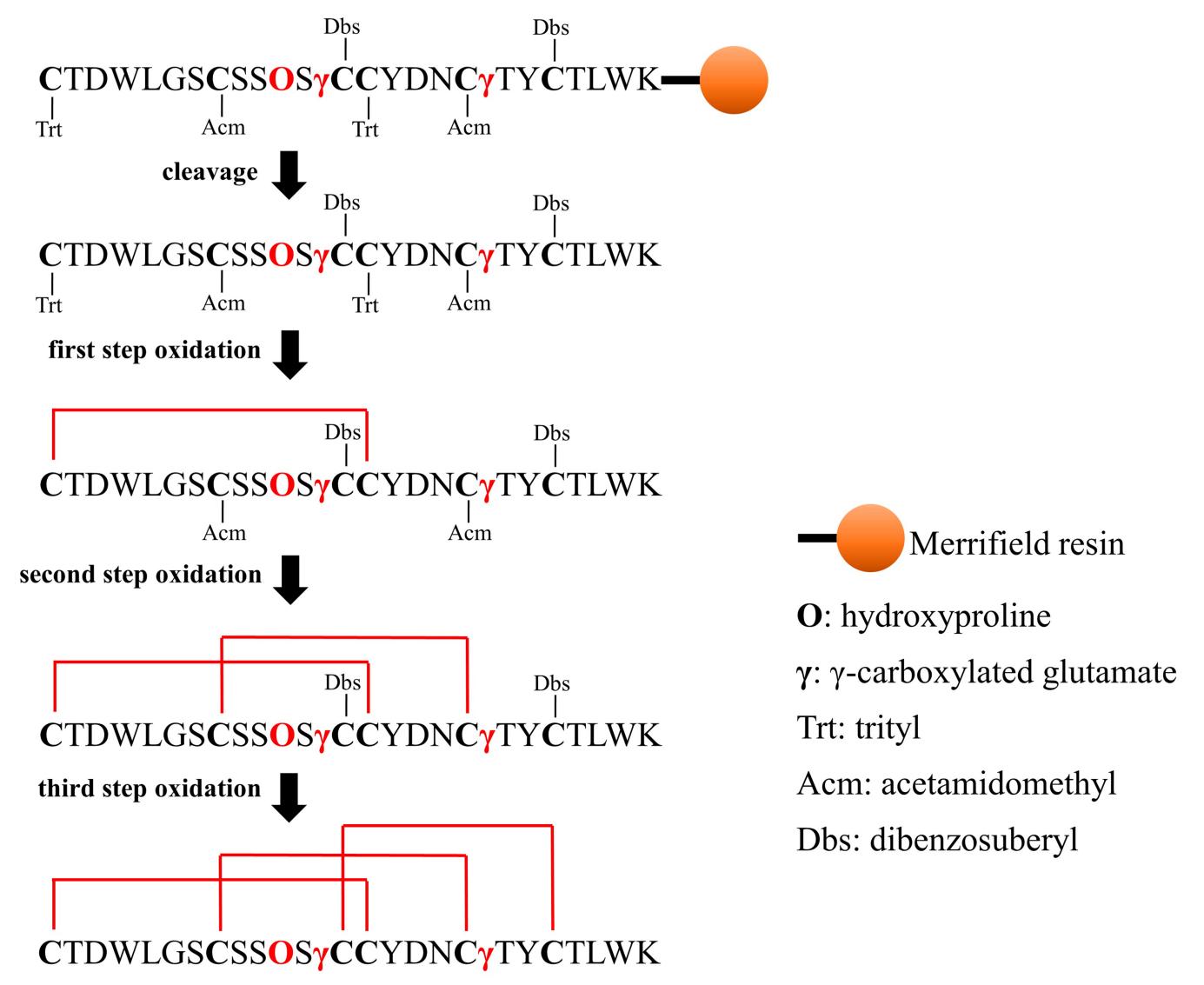
**

**Figure S1.** **Synthetic strategy for peptide Lt7b using solid-phase peptide synthesis.** The three modified amino acids and three disulfide bonds were colored red.

**
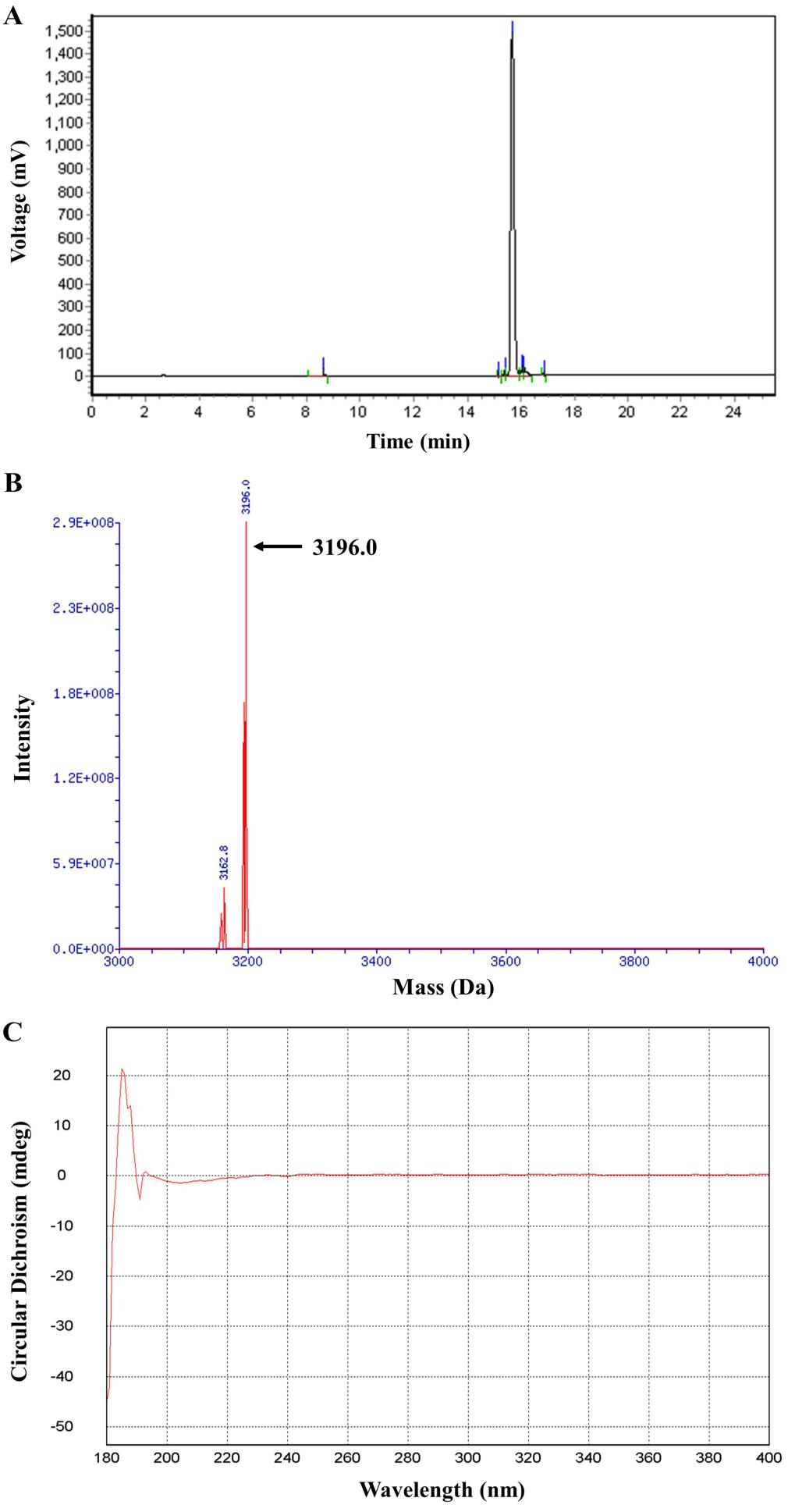
**

**Figure S2. Purification and identification of synthetic Lt7b. (A)** Reverse phase HPLC purification of Lt7b. **(B)** Mass spectra of Lt7b. **(C)** CD spectra of Lt7b.


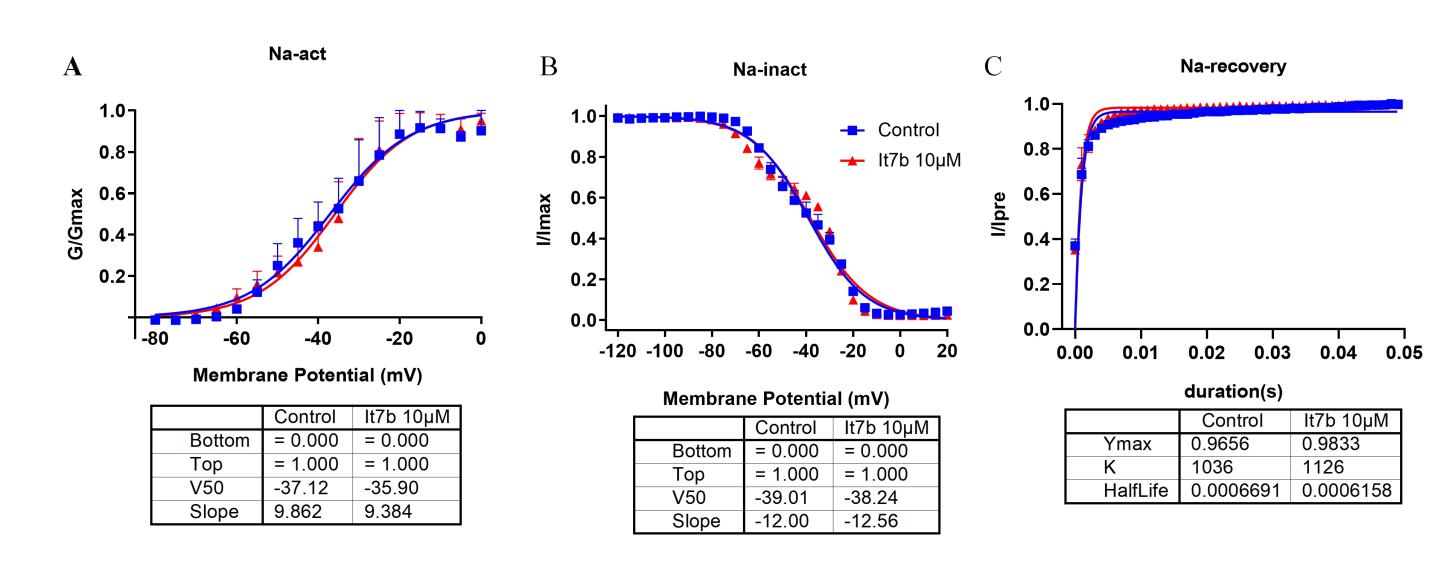


**Figure S3. Effects of Lt7b on sodium currents.** Effects of 10 μM Lt7b on the activation (A), inactivation (B) and recovery (C) of sodium currents in DRG neurons with detailed fit parameters.

**Table S1.** Comparison of several cysteine framework VI/VII O2-conotoxins.

| *Conus* species | Conotoxin | Amino acid sequences | pharmacological targets |
| --- | --- | --- | --- |
| *Conus textile* | TxVIIA | **C**GGYSTY**Cγ**VDS**γCC**SDN**C**VRSY**C**TLF | induced depolarization and increased firing of action potentials in molluscan neuronal systems |
| *Conus pennaceus* | PnVIIA | D**C**TSWFGR**C**TVNS**γCC**SNS**C**DQTY**Cγ**LYAF**O**S |  |
| *Conus litteratus* | Lt7a | GE**C**LGWSNY**C**TSHSI**CC**SGE**C**ILSY**C**DIW | inhibited voltage-sensitive sodium channels |
|  | Lt7b | **C**TDWLGS**C**SS**O**S**γCC**YDN**Cγ**TY**C**TLWK | inhibited calcium currents and increased sodium currents (this study) |
| *Conus princeps* | PiVIIA | **C**DA**O**THY**C**TNYW**γCC**SGY**Cγ**HSH**C**W | increased the Ca^2+^currents |
| *Conus austini* | As7a | T**C**KQKGEG**C**SLDV**γCC**SSS**C**KPGGPLFDFD**C** | undetermined |
| *Conus delessertii* | De7a | A**C**K**O**KNNL**C**AIT**γ**MA**γCC**SGF**C**LIYR**C**S***** | undetermined |

O: hydroxyproline; γ: γ-carboxyglutamate; *: amidated C-terminus.

**Table S2.** Cytotoxicity of Lt7b on HaCaT and ND7/23 cells.

| Cells | Dose (μM) | 0 | 0.01 | 0.1 | 1 | 10 | 100 | 1000 |
| --- | --- | --- | --- | --- | --- | --- | --- | --- |
| HaCaT | OD490 | 1.033±0.070 | 1.092±0.085 | 1.010±0.071 | 1.057±0.081 | 1.032±0.095 | 1.020±0.095 | 0.994±0.097 |
|  | Cell viability (%) | 100 | 105.68 | 97.77 | 102.27 | 99.84 | 98.73 | 96.24 |
| ND7/23 | OD490 | 0.841±0.061 | 0.889±0.065 | 0.846±0.076 | 0.866±0.063 | 0.841±0.089 | 0.825±0.078 | 0.821±0.069 |
|  | Cell viability (%) | 100 | 105.73 | 100.63 | 103.02 | 100.05 | 98.14 | 97.62 |

**3. Supplementary Introduction**

*Conus*, which contains more than 800 cone snail species, is one of the largest genera of marine mollusks [1, 2]. Cone snails can secrete venoms for prey capture or enemy defense [3]. The dominant components of cone snail venoms are small peptides typically comprising 10-50 amino acids and 1-5 disulfide bridges, which are commonly known as conotoxins or conopeptides [4, 5]. Conotoxins are promising neuropharmacology tools and drug candidates due to their high efficiency and specificity in targeting ion channels or neurotransmitter receptors. Conotoxins are used to distinguish different subtypes, discover novel binding sites, and research the structure and functions of the receptors [6]. ω-Conotoxin MVIIA (Ziconotide) was approved by the FDA for treating intractable chronic pain in 2004, and several other conotoxins were under clinical or preclinical phases for the treatment of Alzheimer, Parkinson, epilepsy, chronic pain and cardiovascular diseases [6-8]. Up to date, 2986 nucleic acid sequences, 8123 protein sequences and 222 structures of conotoxins have been collected by ConoServer, a famous database of conotoxins [9].

Based on the highly conserved signal sequence in the precursor, the vast majority of conotoxins can be divided into 29 gene superfamilies. The O2 superfamily was first named in 2006 and mainly includes the cysteine framework VI/VII and XV conotoxins [10-11]. The functions of four cysteine framework VI/VII O2-conotoxins have been identified. TxVIIA and PnVIIA induced depolarization and increased firing of action potentials in molluscan neuronal systems [12, 13]. Lt7a blocked the voltage-sensitive sodium channels in rat dorsal root ganglion (DRG) neurons [14]. PiVIIA selectively increased the Ca^2+^ currents from DRG neurons [15]. Unlike other superfamily conotoxins, O2 superfamily conotoxins have no unified or similar functions, which increases the difficulty of physiological function research.

In this work, we reported the synthesis and physiological functions of a novel O2-superfamily conotoxin, Lt7b, which was first cloned from the venom duct cDNA library of a worm-hunting *Conus literatus* [16]. The precursor of Lt7b comprises 74 amino acid residues, including a mature peptide of 27 amino acid residues (CTDWLGSCSSPSECCYDNCETYCTLWK). Lt7b with three modified amino acids and three disulfide bonds was synthesized by solid-phase polypeptide synthesis and identified by mass spectrum and circular dichroism. Patch clamp on rat DRG cells showed that Lt7b significantly inhibited calcium currents and increased sodium currents. Patch clamp on HEK293 cells showed that Lt7b inhibited the currents of calcium channel subtypes but did not increase the currents of sodium channel subtypes. Moreover, the animal analgesia experiments showed that Lt7b had significant analgesic effects in the hotplate assay. Our results suggested that Lt7b would be a potential lead molecule for the development of new analgesic drugs.

**References**

1. Wu Y, Wang L, Zhou M, You Y, Zhu X, Qiang Y, et al. Molecular evolution and diversity of Conus peptide toxins, as revealed by gene structure and intron sequence analyses. PLoS One. 2013;8:e82495.

2. Himaya SWA, Lewis RJ. Venomics-Accelerated Cone Snail Venom Peptide Discovery. Int J Mol Sci. 2018;19:788.

3. Gao B, Peng C, Yang J, Yi Y, Zhang J, Shi Q. Cone Snails: A Big Store of Conotoxins for Novel Drug Discovery. Toxins (Basel). 2017;9:397.

4. Jin AH, Muttenthaler M, Dutertre S, Himaya SWA, Kaas Q, Craik DJ, et al. Conotoxins: Chemistry and Biology. Chem Rev. 2019;119:11510-11549.

5. Morales Duque H, Campos Dias S, Franco OL. Structural and Functional Analyses of Cone Snail Toxins. Mar Drugs. 2019;17:370.

6. Dao FY, Yang H, Su ZD, Yang W, Wu Y, Hui D, et al. Recent Advances in Conotoxin Classification by Using Machine Learning Methods. Molecules. 2017;22:1057.

7. Yu S, Li Y, Chen J, Zhang Y, Tao X, Dai Q, et al. TAT-Modified omega-Conotoxin MVIIA for Crossing the Blood-Brain Barrier. Mar Drugs. 2019;17:286.

8. Yang M, Zhou M. mu-conotoxin TsIIIA, a peptide inhibitor of human voltage-gated sodium channel hNav1.8. Toxicon. 2020;186:29-34.

9. Kaas Q, Yu R, Jin AH, Dutertre S, Craik DJ. ConoServer: updated content, knowledge, and discovery tools in the conopeptide database. Nucleic Acids Res. 2012;40:D325-330.

10. Zhangsun D, Luo S, Wu Y, Zhu X, Hu Y, Xie L. Novel O-superfamily conotoxins identified by cDNA cloning from three vermivorous Conus species. Chem Biol Drug Des. 2006;68:256-265.

11. Wu Y, Wang L, Zhou M, Jiang X, Zhu X, Chen Y, et al. Soluble expression, purification and functional identification of the framework XV conotoxins derived from different Conus species. Peptides. 2014;56:77-83.

12. Nakamura T, Yu Z, Fainzilber M, Burlingame AL. Mass spectrometric-based revision of the structure of a cysteine-rich peptide toxin with gamma-carboxyglutamic acid, TxVIIA, from the sea snail, Conus textile. Protein Sci. 1996;5:524-530.

13. Fainzilber M, Nakamura T, Lodder JC, Zlotkin E, Kits KS, Burlingame AL. gamma-Conotoxin-PnVIIA, a gamma-carboxyglutamate-containing peptide agonist of neuronal pacemaker cation currents. Biochemistry. 1998;37:1470-1477.

14. Pi C, Liu J, Wang L, Jiang X, Liu Y, Peng C, et al. Soluble expression, purification and functional identification of a disulfide-rich conotoxin derived from Conus litteratus. J Biotechnol. 2007;128:184-193.

15. Bernaldez J, Jimenez S, Gonzalez LJ, Ferro JN, Soto E, Salceda E, et al. A New Member of Gamma-Conotoxin Family Isolated from Conus princeps Displays a Novel Molecular Target. Toxins (Basel). 2016;8:39.

16. Pi C, Liu J, Peng C, Liu Y, Jiang X, Zhao Y, et al. Diversity and evolution of conotoxins based on gene expression profiling of Conus litteratus. Genomics. 2006;88:809-819.

**4. Supplementary Discussion**

In addition to inhibiting calcium currents, 10 μM Lt7b increased the sodium currents by 77.32±8.21% in rat DRG neurons. To the best of our knowledge, two other conotoxins (RXIA and Lt3a) have been reported to increase sodium currents without delayed inactivation. The I1-superfamily conotoxin RXIA can increase the Nav1.6 sodium currents and shift its voltage dependence of activation to more hyperpolarized potentials [1]. The M-superfamily conotoxin Lt3a can enhance tetrodotoxin-sensitive sodium currents in rat DRG neurons [2]. Because the medium DRG neurons (diameters 20–30 μm) that contain both TTX-sensitive and TTX-resistant sodium currents were tested in this study, we further investigated the effects of Lt7b on two rat sodium channel subtypes (TTX-sensitive rNav1.7 and TTX-resistant rNav1.8) and three human sodium channel subtypes (TTX-sensitive hNav1.7, TTX-resistant hNav1.5 and hNav1.8) in HEK293 cells. Unfortunately, no increases were detected in these TTX-sensitive and TTX-resistant sodium currents. One reason may be that Lt7b acts on other rat sodium channel subtypes, such as rNav1.1, rNav1.2, rNav1.3, rNav1.4, rNav1.5 or rNav1.6, which we did not test in this study. Another reason may be that Lt7b acts on other human sodium channel subtypes or even that Lt7b does not act on human sodium channels. The effects of Lt7b on other rat or human sodium channel subtypes need further study. On the other hand, the sodium current increasing activity may introduce negative effects on the analgesic activity of Lt7b, so the detection of no increase on human sodium channel subtype currents may be beneficial to the development of Lt7b as a novel analgesic lead compound.

**References**

1. Buczek O, Wei D, Babon JJ, Yang X, Fiedler B, Chen P, et al. Structure and sodium channel activity of an excitatory I1-superfamily conotoxin. Biochemistry. 2007;46:9929-9940.

2. Wang L, Liu J, Pi C, Zeng X, Zhou M, Jiang X, et al. Identification of a novel M-superfamily conotoxin with the ability to enhance tetrodotoxin sensitive sodium currents. Arch Toxicol. 2009;83:925-932.
